# Preclinical Polymodal Hallucinations for 13 Years before Dementia with Lewy Bodies

**DOI:** 10.1155/2014/694296

**Published:** 2014-04-27

**Authors:** Carlo Abbate, Pietro Davide Trimarchi, Silvia Inglese, Niccolò Viti, Alessandra Cantatore, Lisa De Agostini, Federico Pirri, Lorenza Marino, Renzo Bagarolo, Daniela Mari

**Affiliations:** ^1^Fondazione IRCCS Ca' Granda, Ospedale Maggiore Policlinico, Unità Operativa Complessa di Geriatria, Via Pace 9, 20122 Milan, Italy; ^2^Fondazione IRCCS Don Carlo Gnocchi, ONLUS, S. Maria Nascente, Via Capecelatro 66, 20148 Milan, Italy; ^3^Dipartimento di Scienze Cliniche e di Comunità, Università degli Studi di Milano, Via Della Commenda 9/12, 20122 Milan, Italy

## Abstract

*Objective*. We describe a case of dementia with Lewy bodies (DLB) that presented long-lasting preclinical complex polymodal hallucinations. *Background*. Few studies have deeply investigated the characteristics of hallucinations in DLB, especially in the preclinical phase. Moreover, the clinical phenotype of mild cognitive impairment-(MCI-) DLB is poorly understood. *Methods*. The patient was followed for 4 years and a selective phenomenological and cognitive study was performed at the predementia stage. *Results*. The phenomenological study showed the presence of hypnagogic and hypnopompic hallucinations that allowed us to make a differential diagnosis between DLB and Charles Bonnet syndrome (CBS). The neuropsychological evaluation showed a multiple domain without amnesia MCI subtype with prefrontal dysexecutive, visuoperceptual, and visuospatial impairments and simultanagnosia, which has not previously been reported in MCI-DLB. *Conclusions*. This study extends the prognostic value of hallucinations for DLB to the preclinical phases. It supports and refines the MCI-DLB concept and identifies simultanagnosia as a possible early cognitive marker. Finally, it confirms an association between hallucinations and visuoperceptual impairments at an intermediate stage of the disease course and strongly supports the hypothesis that hallucinations in the earliest stages of DLB may reflect a narcolepsy-like REM-sleep disorder.

## 1. Introduction

### 1.1. Hallucinations in Dementia with Lewy Bodies

Dementia with Lewy bodies (DLB) is the second most frequent type of neurodegenerative dementia after Alzheimer's disease (AD). Complex visual hallucinations are common symptoms in DLB [1–3] and have been identified as one of the core features of DLB in current diagnostic criteria [4]. Hallucinations in different modalities represent a supportive feature in diagnostic criteria. Visual hallucinations are the best positive predictor of DLB at autopsy and, together with visuospatial constructional impairments, represent the best model for differentiating DLB from AD at the earliest stages of disease [5]. However, few studies have deeply investigated the characteristics of visual hallucinations in DLB [1, 6, 7]. Sometimes hallucinations can be the unique early sign of DLB without any other associated psychotic, motor, or cognitive symptoms, especially in the early stages of dementia [8, 9]. In these DLB cases, the differential diagnosis with other pathological conditions causing hallucinations in the elderly, such as Charles Bonnet syndrome (CBS), can be somewhat difficult. CBS is a common cause of vivid, formed, complex, and persistent visual hallucinations in otherwise psychologically normal elderly [10, 11]. The possibility that patients with DLB could be misdiagnosed with CBS, particularly in the early stages, had been previously hypothesized [12], and careful longitudinal observations are recommended to confirm the presence of DLB in elderly patients with suspected CBS [8, 12]. In fact, some authors suspect that CBS cases described in the literature [13, 14] are in fact DLB cases [15, 16], and there are reports of some true DLB cases being mistaken for CBS in the early phase of dementia [8, 9]. These last mistakes are most likely related to the fact that the early clinical manifestations of DLB and, in particular, of the mild cognitive impairment (MCI) phase of DLB are poorly defined.

### 1.2. Mild Cognitive Impairment-DLB

MCI refers to the clinical condition of a person who suffers from slight cognitive deficits while preserving the ability to perform the activities of daily living [17]. Research has shown that patients with MCI have an increased risk of developing dementia, and MCI is considered to be a transitional stage along the cognitive continuum from normal aging to overt dementia [18, 19]. The clinical presentation of MCI is heterogeneous and at least 4 subtypes of MCI have been proposed in relation to the profile of cognitive deficits: pure amnesic MCI (aMCI), multiple domains plus amnesia MCI (mdMCIa), multiple domains without amnesia MCI (mdMCIna), and single domain different from memory MCI (snmMCI) [20]. There is emerging evidence that different MCI subtypes can convert to different types of dementia [21, 22]. Some patients with DLB are found to pass through an MCI stage [9, 23]. However, as stated above, the clinical phenotype of MCI-DLB is poorly defined [23]. Some data support that any MCI subtype is possible in DLB, but a multiple domain without amnesia MCI subtype seems to be the most typical [9]. A cognitive profile characterized by attentive/executive prefrontal and visuospatial/constructional impairments is consistently reported, with memory and language functions being more variably affected and often relatively preserved [9, 24].

### 1.3. Account of the DLB Hallucinations

Impairments of visual perception and visual attention are usually reported in the dementia phase of DLB [25–27]. Moreover, DLB patients with visual hallucinations are more impaired in visual perception compared to DLB patients without hallucinations [27]. Thus, visual hallucinations in DLB are related to visuoperceptual function impairments [26, 27].

A different account for DLB visual hallucinations assumes that this phenomenon, together with other DLB symptoms (i.e., hypersomnolence, REM behavior disorders (RBD), etc.), may reflect a narcolepsy-like REM-sleep disorder [28, 29]. Hallucinations in narcolepsy are vivid sensory experiences, including visual, tactile, kinetic, and auditory phenomena, that occur during transitions into (hypnagogic) or out of (hypnopompic) sleep [30, 31]. They are considered to be intrusions of dream imagery into wakefulness [29].

This paper reports the case of an elderly patient (BG) with a clinical diagnosis of probable DLB, presenting a long preclinical phase of isolated complex polymodal hallucinations and a predementia phase of MCI-DLB. BG has been followed up longitudinally for 4 years, and a detailed study of the phenomenology of her hallucinations and the cognitive functions was performed at the predementia stage. The aims of this study are (i) to further highlight the difficulties in a differential diagnosis of complex hallucinations in an elderly patient and to evaluate the possible contributions to differential diagnoses from the study of phenomenology of hallucinations, (ii) to discuss the current accounts of DLB-hallucinations in light of the findings from the BG case, and (iii) to offer data supporting and further refining the concept of MCI-DLB.

## 2. Methods

### 2.1. Case Report

**Figure 1:**
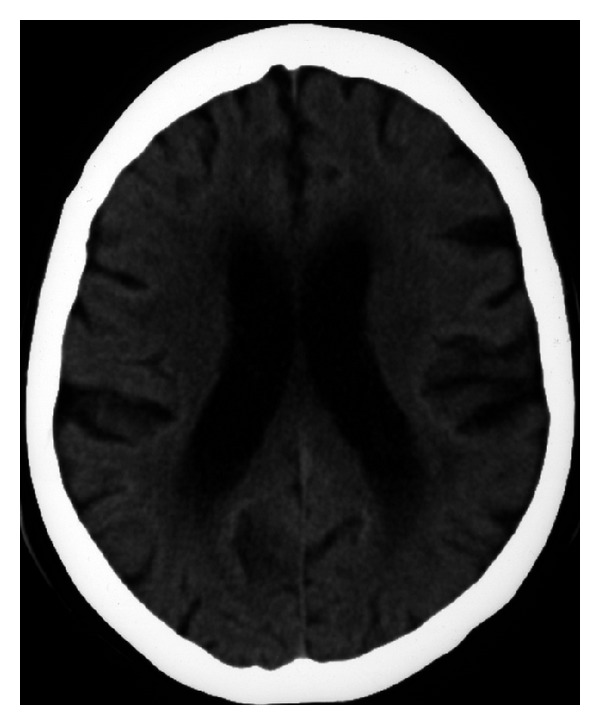
Brain CT-scan performed during follow-up in 2008, showing periventricular white matter damage (left-right was inverted as radiological convention).

We first visited the patient BG in July 2004, when she was 90 years old. She was a right-handed woman, with 5 years of education, and she was formerly a seamstress. She had one daughter and had been a widow for 10 years. BG lived alone in a flat, with a good level of autonomy in her daily living. She had no previous history of severe neurological or psychopathological disorders, alcoholism, or medical conditions, which could potentially cause cognitive impairment or psychiatric symptoms. She had never smoked and now suffered from mild hypertension. Her family history was positive for ischemic heart attack and stroke and negative for dementia. BG had no visual or auditory sensory impairments. During the visit, albeit reluctantly, she referred to a history of frequent complex hallucinations that started approximately 10 years previously, precisely, on June 2, 1994, when she was nearly 80 years old. She was able to be accurate in dating the onset of hallucinations because the second day of June is the Italian Republic anniversary, and exactly three months before, on March 2 of the same year, her husband had died. Hallucinations had been particularly evident during the last two years and had increased in frequency over the ten months before referral. BG, mainly, when relaxed on her bed before sleeping or during the night, saw people, heard their voices, or felt herself touched by them. These people were mostly unfamiliar adults. At other times, her husband or a pretty round-headed child appeared inside a see-through case. The phenomena lasted only few seconds or few minutes and made BG alternately surprised and curious or annoyed and irritated. The “wandering souls,” in BG's words, inhabited her house and appeared mostly in her bedroom, inside the pillow or the mattress, and in the bathroom. People usually spoke aloud or whispered to her and to each other, attended to her, hit her body as she lay down on the bed, or made some grooves in the bed. The patient and her daughter did not report cognitive impairments or other neurological symptoms aside from the hallucinations. A neurological exam was fully negative. No cognitive impairment emerged on a mini mental state examination (MMSE = 29/30) [32], and no signs of depression appeared on the Geriatric Depression Scale (GDS = 5/30) [33]. Blood tests were normal, and the Hachinski scale score [34] was 3 points. A CT-scan revealed mild diffuse white matter vascular damage with leukoaraiosis and small, disseminate ischemic microlesions as well as very mild cortical atrophy. At this point, we were not able to make a definite diagnosis; there were different hypotheses regarding the origin of the complex hallucinations, and they all seemed acceptable (e.g., functional psychiatric illness, CBS, preclinical vascular dementia with psychosis, preclinical DLB, etc.). To improve the case study, we planned to analyze the phenomenology of hallucinations and to deeply examine the cognitive profile. Thus, in the aftermath, we interviewed BG and performed a selective neuropsychological assessment. Because some slight cognitive deficits without a diffuse cognitive decline had been highlighted in a neuropsychological evaluation, we made a diagnosis of MCI of unknown etiology. No therapy for hallucinations was administered because BG did not seem to be very distressed by them. The clinical picture remained stable over three years as shown by subsequent follow-up examinations, until October 2007, when we suddenly and quickly registered a deterioration of cognitive functions (see Table 1). Her MMSE score was 25 points in October, and after only two months, in December, it was 18 points. Her autonomy in daily living had become severely impaired (activities of daily living scale, ADL = 3/6; instrumental activities of daily living scale, IADL = 1/8) [35, 36]. In January 2008, her MMSE score was 15, and a further neuropsychological evaluation showed severe temporal and spatial disorientation, attention impairments, a dysexecutive prefrontal syndrome, and reduced insight. Anterograde and retrograde amnesia with temporal transpositions of recalled episodes and autobiographical dysmnesia were also present. Conversational speech showed signs of dysarthria and dysphasia, with slow articulation, word-finding problems, and some phonological paraphasias. Moreover, BG's speech was often confused, and we noticed auditory comprehension difficulties. Relatives referred dressing apraxia. Moreover, constructional apraxia with closing-in phenomena was revealed by a psychometric test. A complex delusional misidentification syndrome emerged, with simple misidentifications of her relatives; Capgras syndrome with reduplication involving her daughter; misidentification of her own self in the mirror; delusions of inanimate objects, mostly concerning furniture; and, finally, signs of reduplicative paramnesia involving her home. Other types of delusions also emerged; for example, BG hid money and jewelry because she was frightened that the double of her daughter might steal them. Finally, psychomotor slowness and remarkable cognitive and attentive fluctuations were apparent. Behavioral assessment revealed purposeless motor activity, akathisia, and hostility. Relatives did not report any symptoms suggestive of RBD. Complex hallucinations, after being present for over 13 years, had now completely ceased. Occasionally, BG remembered people who had been the subjects of her hallucinations and addressed them as “those who disturbed me,” but she said that she had no longer “seen” them or “listened” to their voices. A neurological exam revealed a rigid-akinetic parkinsonism, with marked bradykinesia and bradyphrenia, mild rigidity, and increased muscular tone. A follow-up CT-scan confirmed mild diffuse cerebral vascular damage to the white matter (periventricular damage and small, dispersed ischemic microlesions), with no new ischemic lesions, and revealed more pronounced cortical and subcortical atrophy (Figure 1). Blood tests were normal. At this point, when BG was 93 years old, dementia was evident. We made a diagnosis of probable DLB, according to known criteria [4] and suggested therapy with rivastigmine. In May 2008, BG's MMSE score was 10 and there was a further reduction of autonomy in daily living (ADL = 2/6 and IADL = 1/8). Further evaluations were no longer possible because of severe dementia.

### 2.2. Phenomenological Study of the Hallucinations

The aim of this section was to study and describe the phenomenology of BG's hallucinations. The analysis was based on a semistructured, open-ended interview held in October 2004. The interview was tape-recorded and transcribed. We investigated a range of attributes of the hallucinations, referring to the episodes (number, duration, and frequency), to the experience of the hallucinations (sensory modality, content, type, clarity, presence of colors, stereotyped, recurrent objects, influence of eyelids, moving with eyes, movement “en bloc,” and intrinsic movement), to the patient (awareness of the unreal nature of phenomena, ease of correction, personal relevance, familiarity of content, and emotional impact), and to the surroundings (relationships with surroundings and circumstances that seem to favor or stop hallucinations), as suggested by other authors [10]. BG was asked for and gave her informed consent before interviewing and testing.

### 2.3. Neuropsychological Assessment

Global cognitive functioning was measured by the MMSE [32], and general intellectual functions were tested with* Raven's colored progressive matrices* [37]. Executive functions were evaluated using the* Wisconsin card sorting test* (WCST) [38], a switching task (*trail making test*, TMT, part B) [39], a categorical thinking task (*Weigl's sorting test*) [40], the* cognitive estimate test* (CET) [41], both a semantic [40] and a phonological verbal fluency task [42], and a working memory task (*digit span backward*) [43]. A verbal short-term memory was rated with the* digit span forward* [43] and visuospatial short-term memory with* Corsi's block tapping test* [40]. Anterograde memory (recall) was tested with delayed recall tasks, both visual and verbal (*prose recall* and* Rey-Osterrieth complex figure recall test*) [40, 44], with (learning) the* paired associated words learning *test [45] and a visuospatial learning test (*Corsi supraspan test*) [40]. Lexical and semantic memory abilities were studied with a vocabulary test (taken from the* Confabulation Battery*) [46] and a picture-to-picture and word-to-word matching task [47]. We tested visual attention with* Bell's test* [48] and a digit cancellation test [40] and measured psychomotor speed with TMT (part A) [39]. Language was examined with the Italian version of the Aachener aphasia test [49] and with a* picture naming task* [50]. Limb [51] and oral apraxia tests [52] were also administered. Visual perception was evaluated using the* discrimination of scribbles test* [40], three tasks (*minimal feature view task*,* object decision task*, and* associative match task*) taken from the* Birmingham Object Recognition Battery* (BORB) [53], and object recognition performance on a* picture naming task* [50]. A finger localization test was administered for assessing finger agnosia [40]. Visuospatial and constructional abilities were explored with a* dot counting* task taken from the* Visual Object Spatial Perception* battery (VOSP) [54] and by means of the* copy of geometrical figures test* [40] as well as the* copy of the Rey-Osterrieth complex figure test* [44]. An* ad hoc* version of* Navon's letters* task was administered to BG and also to a small group of healthy control participants (*n* = 6) to assess simultanagnosia. The mean age of the control group was comparable to BG (mean = 88.83, SD = 4.74, range = 85–99 years), while education was slightly superior (mean = 10.5, SD = 4.75, range = 5–18). All control subjects demonstrated no severe visual impairments or dementia (MMSE mean performance = 28.67, SD = 1.03, range = 27–30). Test material consisted of 10 large alphabetic letters, each shaped by many reproductions of a different small letter. The letters were presented one at a time on separate blank sheets of paper, and this procedure was repeated three times. One point was scored for each letter (10 large and 10 small) recognized, with a partial score of 20 points maximum for each session. The total score range was 0–60. Crawford's statistical procedures for single-case analysis were adopted to compare BG's performance to that of the control subjects [55, 56]. Control subjects gave their written consent to the study.

## 3. Results

### 3.1. The Study of the Hallucinations

#### 3.1.1. Episodes

BG reported 22 distinct episodes of hallucinations during the interview (see Table 2). Episodes appeared with a moderate-high frequency, daily or at a time interval of one/two days. The duration was usually brief: seconds, tens of seconds, or a maximum of several minutes. In two episodes, the patient referred to a hallucination that lasted for many hours.

#### 3.1.2. Experience of Hallucination

Hallucinations were polymodal, integrating auditory (verbal and not verbal), visual, and somatosensory (tactile, kinesthesic) perceptions. They were mostly complex but in some minor cases, especially for the somatosensory channel, quite simple (i.e., vibrations at the foot of the bed, hits on the back, etc.). The contents were people and people in miniature, voices and whispers of people, simple rumors, vibrations or movements, tactile and motion sensations, and a little bit of light. Hallucinations were both hypnagogic and hypnopompic. Two episodes were clearly lilliputian. Most of the episodes happened during the late evening (usually from 8.30 p.m. to 9.30 p.m.), when the patient was going to sleep and was often lying down on bed, relaxed, and most likely with her eyes closed. Many episodes occurred in the middle of the night, most likely when the patient awakened and opened her eyes. Four episodes occurred during the day, when the patient was totally awake and open-eyed. Hallucinations usually possessed good clarity, with the same fidelity as reality. They occurred in black and white and were rarely colored; in only one episode, BG described a red-haired woman. Sometimes the sensations of specific objects had returned (i.e., her husband's voice) and a few hallucinations were stereotyped (i.e., vibrations at the foot of the bed). However, in most of the episodes, objects were new and original. Generally, her eyelids did not seem to influence the phenomena. On only two occasions, BG stated that opening her eyes stopped the hallucinations. Hallucinations showed intrinsic movements in some episodes and were motionless in others. There was no evidence of a relationship between movements of images and eye movement. Other phenomena seemed to be associated with BG's hallucinations. In particular, BG believed that the unfamiliar people of her hallucinations were living in her house, a false belief resembling the phantom boarder symptom (PBS) [57, 58]. Moreover, the feeling of a presence [59] was sometimes associated with hallucinations involving her husband.

#### 3.1.3. Patient

BG appropriately considered her hallucinations as extraordinary phenomena, and she was embarrassed and reluctant to mention her experiences to relatives and clinicians, fearing that she would be considered insane. Otherwise, BG was not aware of the unreal nature of her hallucinations. Much evidence supported this conclusion: she often conversed with the person in the hallucinations and performed concrete actions aimed to either not disturb them (i.e., she replaced her husband's photograph on her night table with a photo of Our Lady of Fatima because her hallucinations did not like it; she turned off the fan despite extreme heat because her hallucinations did not like the fan because they were so light) or to prevent their return (i.e., she rolled her pillows tight so that her husband could not get into them; she wore rosary beads sprinkled with holy water on the bed to keep them away). BG was never corrected by others while hallucinating. The content was both familiar with a high personal relevance (i.e., her husband's voice) or unfamiliar with no personal relevance. The emotional impact was both positive and negative and was neutral in only a minority of episodes. Positive feelings were mainly happiness, surprise, and curiosity. BG was happy and surprised, especially when her husband was there. Positive reactions were provoked also by unknown people who smiled or did something to help her on many episodes (i.e., one of them pointed the way to the bathroom during the night; another one seemed to help her with the plug; an old man seemed to put a lid on her; and her husband informed her about the black out and the evacuation for a bomb). On one occasion, BG stated that she felt affection for one of the unknown people in her hallucination because he did not hurt her and kept her company. Negative reactions were mainly annoyance, a feeling of being bothered, tension, and discomfort but never fear, hate, hostility, or belligerence. BG clearly felt dismay on only one occasion. Mainly she became annoyed by noises and vibrations in her bedroom and felt embarrassed because the people of hallucination inhabited her bed, and she had the impression that they did not take their eyes off of her. Other times she became annoyed because she felt forced to perform some actions aimed at not disturbing them.

#### 3.1.4. Surrounding

The people and objects of hallucinations always fit well within the surrounding. BG was not able to condition the content of her hallucinations. On one occasion, BG stated that turning on a light stopped the hallucinations. Generally, she did not explicitly report circumstances that favored the occurrence of hallucinations or methods to stop them.

### 3.2. Neuropsychological Assessment

**Figure 2:**
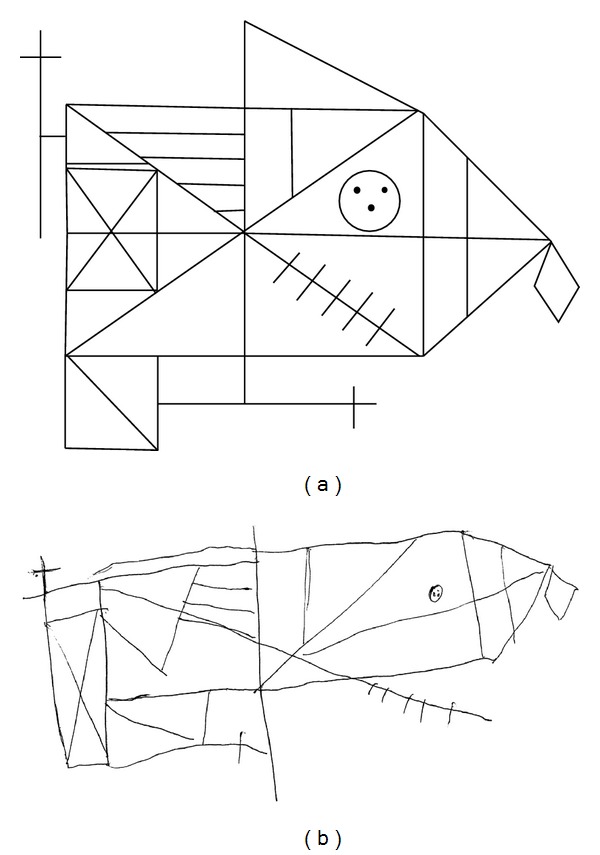
BG's copy of the Rey-Osterrieth complex figure.

As shown in Table 3, global cognitive functioning and general intellectual functions were preserved. A slight impairment of prefrontal executive functions emerged. In particular, BG had some difficulty on the WCST [38] and her performance was impaired on the switching task (TMT, part B) [39], on the categorical thinking task (Weigl test) [40], and on the CET [41]. Her performance on the other prefrontal tests, the semantic and phonologic verbal fluency tasks and the working memory task, was normal. A slight psychomotor slowness was evident, as measured by the tracking task (TMT, part A). Short-term, anterograde, and semantic memory were all preserved. No impairments of selective visual attention, visual spatial attention (neglect), language, ideomotor, and orofacial praxis emerged. A significant impairment resulted on the object decision task from the BORB [53], suggesting an isolated mild disruption at the structural description stage of visual processing [60]. No other impairments of visual perception from the early (discrimination of scribbles test and minimal feature view task from BORB) to late (picture naming test and associative match task from BORB) stages of visual processing emerged. A significant deficit, suggesting simultanagnosia, appeared on Navon's letters task (BG's performance = 30/60; mean control performance = 52.5, SD = 6.5; *t* = −3.205, *P* = .024, two-tailed). A mild visuoconstructional impairment resulted from the copy of Rey-Osterrieth complex figure test [44] (see Figure 2). The copy of geometrical figures test was globally unimpaired; nevertheless, BG made two errors while drawing the two most complex figures included in this test. No impairments of other visuospatial abilities emerged (dot counting task). Finally, no signs of finger agnosia emerged on the finger localization test [40].

## 4. Discussion

### 4.1. DLB Diagnosis

BG's complete medical case, including both the long preclinical phase of complex hallucinations and the phase of full blown dementia, supported a clinical diagnosis of probable DLB, according to known criteria [4, 61]. The central (dementia) and all three core features (fluctuations, parkinsonism, and hallucinations) of DLB were present. Moreover, hallucinations in several sensory modalities and systematized delusions (in BG's case, characterized by a complex delusional misidentification syndrome, an element frequently reported in the literature [2, 3, 62]) added two supportive features to the DLB diagnosis. Finally, even if the extreme rapidly worsening of dementia lasting for a few months was not included in diagnostic criteria, this feature has been reported in other DLB cases [63]. Although clinical signs and symptoms suggest a DLB diagnosis, unfortunately, we have no further brain imaging (i.e., PET imaging, SPECT dopamine transporter imaging) or neuropathological data to support it [4, 61], and this is a limitation of the present case report.

A diagnosis of vascular dementia with psychosis was a valid alternative hypothesis at the first interview. Certainly, the medical history of BG was negative for stroke or other vascular accidents, and her Hachinski ischemic score was negative for vascular dementia. Nonetheless, she suffered from mild hypertension for a few years, and her family history was positive for ischemic heart attack and stroke. Most importantly, a CT-scan showed diffuse periventricular damage (leukoaraiosis) and small, disseminate ischemic microlesions. Moreover, past studies revealed that some patients with vascular dementia, especially of the subcortical type, have hallucinations in the early stages [64–66]. Finally, BG's cognitive impairment profile at the MCI stage was compatible with the early cognitive syndrome of subcortical vascular dementia, which is characterized by an impairment of attention and executive functions with the slowing of motor performance and information processing [67, 68]. Nonetheless, we rejected a diagnosis of subcortical vascular dementia because it failed to explain the full clinical presentation. First, a severe dementia syndrome is uncommon in subcortical vascular dementia [67], while its cardinal manifestation is a subcortical cognitive syndrome attributed to preferential damage to the prefrontal-subcortical circuits, with higher cortical functions preserved [67–69]. On the contrary, BG's dementia was characterized by severe and multiple cognitive impairments, suggesting diffuse cortical damage (i.e., retrograde and autobiographical amnesia, aphasia, constructional and dressing apraxia, a complex misidentification syndrome, etc.). Second, hallucinations in the early stages of subcortical vascular dementia do not usually involve symptoms that occur during sleep induction or awakening, such as the preferential hypnagogic and hypnopompic hallucinations experienced by BG [65]. Third, the clinical picture of subcortical vascular dementia involves widespread manifestations, including cognitive impairments, personality and mood disorders (i.e., apathy, irritability, and vascular depression), psychotic symptoms, gait disturbances, motor dysfunction, and urinary symptoms [67, 69]. BG's clinical picture lacks many of these symptoms and is characterized by isolated hallucinations for a very long period. Finally, the evidence of cerebrovascular disease is not incompatible with a diagnosis of DLB, considering that white matter lesions, microvascular changes, and lacunae may be present in up to 30% of autopsy-confirmed DLB cases [4].

### 4.2. Differential Diagnosis of Hallucinations

We decided to perform a detailed study of BG's hallucinations to better understand this case. In particular, we believed that such a study could be a valuable aid for differential diagnosis, especially for BG's case and other similar cases where hallucinations were the unique, isolated, early symptom [8, 9]. The results of the study showed that the polymodal hallucinations experienced by BG effectively exhibited some of the main features described in the few studies addressing the phenomenology of hallucinations in DLB [6, 7]. Hallucinations were multiple and recurrent, and BG frequently heard her hallucinations while speaking [6]. Moreover, hallucinations were complex and experienced daily; they involved a single object perceived in the central visual field, rather than extended scenes, and in the peripheral visual field [7]. Hallucinations persisted with closed eyes and rarely moved with eye movement; they were constant rather than changing from one thing into another, and they were usually opaque and were only transparent in a few cases [7]. Visual and auditory hallucinations are the most common modalities in DLB, similar to Parkinson's disease and Parkinson's dementia; however, olfactory and tactile hallucinations may also be present in some cases [1].

However, different from the more common phenomenology of DLB hallucinations, BG's hallucinations lasted seconds or tens of seconds rather than minutes, they were generally in black and white and rarely in color, and they showed intrinsic movement. Moreover, DLB patients usually find their hallucinations unpleasant [7], while BG experienced both positive and negative reactions.

We also considered the hypothesis that BG might suffer from CBS. Many characteristics of BG's hallucinations were different from typical CBS cases [10, 11]. CBS patients are often fully or partially aware of the unreal nature of their hallucinations and are always easily corrected by others while hallucinating [10]. On the contrary, BG lacked this insight and it was never possible to correct her while hallucinating. Hallucinatory experiences of CBS are exclusively or primarily visual [11]. On the contrary, BG's hallucinations were polymodal (visual, auditory, and somatosensory), with no sensory modality prevailing over the others. Normal consciousness is characteristic of CBS, whereas BG experienced overall hypnagogic and hypnopompic hallucinations that commonly occurred when the patient was drowsy [16]. Finally, even if a visual dysfunction was not mandatory for a CBS diagnosis [11], the majority of CBS patients, unlike BG, have severe visual impairments. All the above evidence was not consistent with a CBS diagnosis and led us to reject the CBS hypothesis.

At first, we did not exclude a psychopathological basis for BG's hallucinations, considering that the hallucinations started in a condition of loneliness and in a reactive depressive mood state just three months after BG's husband died. However, there was not a history of psychiatric disease and there were no other psychiatric symptoms associated with the hallucinations (i.e., delusions, paranoid ideations, etc.), excluding some inconstant evidence for PBS [57, 58] and the feeling of a presence [59]. Our study of BG's hallucination phenomenology evidenced some features different from those that usually characterize psychiatric visual hallucinations [70] and, for that reason, we rejected this possibility.

Peduncular hallucinosis (PH) is characterized by striking visual images, which may persist for years, very similar to the hypnagogic hallucinations experienced by BG. PH most commonly has a vascular aetiology [71, 72]. Thus, we considered PH as a possible diagnosis. However, PH is only occasionally accompanied by tactile and auditory hallucinations and the hallucinations often last more than tens of seconds and are experienced during normal wakefulness, and the patients usually have insights into their hallucinations [71]. Moreover, hypersomnolence and oculomotor disturbances are often present [73].

Vascular etiology was another possible cause of BG's complex hallucinations. Focal vascular insults to the posterior circulation can be associated with complex visual and multimodal hallucinations in the elderly [74, 75]. However, in these cases, hallucinations are typically limited to the abnormal visual field [71] and appear to arise from the opposite side of the stroke [74]. BG's hallucinations had none of these features. Moreover, as already reported, early hallucinations can be present in subcortical vascular dementia but usually do not involve hypnopompic and hypnagogic phenomena [65].

In conclusion, the study of BG's hallucinations has provided some interesting clues about BG's abnormal experience and offered a contribution to her differential diagnosis. Moreover, it allowed us to recognize a common unique feature or a stable collection of key elements that could reliably distinguish DLB hallucinations from hallucinations caused by other pathologies, serving as an early marker of DLB hallucinations. However, at present, even if some common features of DLB hallucinations have been identified, relative heterogeneity among different patients remains and few DLB patients have been studied accurately [7].

### 4.3. Account of the DLB Hallucinations

Visual hallucinations in DLB have been related to visuoperceptual function impairments [26, 27]. Because the phenomenology of DLB hallucinations included colors, detailed objects, and people as well as motion and scenery, some authors have suggested that both visual pathways of higher visual processing, the ventral and the dorsal pathways, are likely involved [7]. Some studies have supported an association between perception impairment and hallucinations in DLB patients, involving brain perfusion modifications in various cerebral areas that are usually involved in visual processing [76–78]. In particular, some authors found a significant relationship between decreased hallucination scores and increased perfusion in the midline parietal area [76]. In another study, both visual hallucinations and occipital hypoperfusion increased with donepezil therapy in DLB, suggesting that occipital cholinergic deficits, occipital hypoperfusion, and visual hallucinations may be directly interrelated [77]. Finally, a significant hypometabolism was found by other authors in visual association areas (i.e., the right occipitotemporal junction and the right middle frontal gyrus) of DLB patients with visual hallucinations [78]. More recently, a significant hypoperfusion in both the ventral visual stream for face and object recognition (i.e., left ventral occipital gyrus) and in the dorsal visual stream for spatial location and motion vision (i.e., bilateral parietal cortices) has been confirmed in DLB patients with hallucinations [3]. BG's case study offers data that support the hypothesized association between visual hallucinations and visuoperceptual impairment in DLB [26, 27] and that support the hypothesis suggesting that both the ventral and dorsal pathways of higher visual processing are involved [7]. In fact, BG showed both a visuoperceptual deficit, most likely at the structural description stage of visual processing, and a visuospatial disorder with constructional impairments and (dorsal) simultanagnosia. However, BG's polymodal hallucinations (visual, auditory, and somatosensory) were quite unusual compared to the typical, predominantly visual hallucinations in DLB. Thus, the BG case could be viewed as atypical.

Actually, BG's hallucinations most likely started several years before any cognitive impairment. Thus, BG's case could argue against an explanation of DLB hallucinations based exclusively on the association of perceptual versus spatial cognitive impairments. On the contrary, the study of BG's hallucinations, showing almost exclusive hypnagogic and hypnopompic phenomena, could support the view that hallucinations associated with DLB, especially in the early stages of the disease, may reflect a narcolepsy-like REM disorder [28, 29]. Interestingly, RBD, a REM-sleep-related phenomenon that sometimes is a narcolepsy symptom, can be an early marker of DLB, presenting as the unique symptom for several years before the onset of dementia, just like BG's preclinical hallucinations did [29, 79–81]. The temporal sequence of pathology postulated in PD [82, 83] and in DLB [84]—from the medulla upwards through the brainstem, to the substantia nigra, the limbic structures including the amygdala, and finally the neocortex—is consistent with preclinical RBD and hypnagogic hallucinations preceding the onset of parkinsonism and cognitive impairments in some DLB patients [80]. Pontomedullary nuclei in the brainstem involved in early Lewy pathology include the perilocus ceruleus regions [82, 83], which some authors have found to be the RBD substrate (subceruleus) and the possible substrate (preceruleus) for hypnagogic hallucinations in rats [85].

### 4.4. The MCI-DLB Concept

Our case study replicates the findings obtained by other authors that hallucinations can be the unique early sign of DLB without any other associated psychotic, motor, or cognitive symptoms [8, 9]. We did not know if MCI was present when hallucination started in 1994, but we considered this eventuality as unlikely. In fact, we detected MCI incidentally in 2004, when we decided to administer a complete neuropsychological evaluation that was planned to improve our knowledge about a strange case of an elderly patient presenting complex enduring hallucinations. Considering this ten-year interval between hallucination onset and MCI diagnosis, and an MCI duration of approximately 3 years before dementia onset, it is reasonable to think that MCI began sometime after the hallucinations. Furthermore, some authors found a trend for a shorter duration of MCI-DLB compared to MCI-AD, suggesting a more rapid course for MCI-DLB [23]. In general, BG's case study confirms once more the relevance of hallucinations to a DLB diagnosis [2, 4] and extends their prognostic value from the early stages of disease [5] back to the preclinical phase. Moreover, our case study is consistent with several other DLB cases showing long courses, with preclinical periods of hallucinations [8, 9], RBD [29, 79–81], or dysautonomia [86, 87] extending back several years, or decades, in at least some cases [81].

Overall, our case study offers further data supporting the concept of MCI-DLB [9, 23, 24]. Moreover, BG's multiple domain, nonamnestic MCI subtype has been already reported in other DLB cases [9]. According to the typical cognitive profile of DLB in the early stages of dementia [5, 25] and in MCI cases [9], the main features of BG's cognitive profile were attentive/executive prefrontal and visuospatial/constructional impairment, with a good preservation of memory, language, and praxis. These impairments are also known to be prominent features of the DLB cognitive profile during the dementia phase [88–92]. The slight psychomotor slowness showed by BG was also an MCI-DLB aspect already reported [23].

Interestingly, simultanagnosia was an early and striking feature of BG's cognitive profile. Our evaluation was not directly aimed at distinguishing between the ventral and dorsal types of simultanagnosia [93]. Nonetheless, BG showed normal speed in reading and no tendency to read letter-by-letter as sometimes manifested by ventral simultanagnosia [94]. Thus, our data suggest, in a preliminary fashion, that BG could suffer from dorsal simultanagnosia. To our knowledge, this is the first report of simultanagnosia in a MCI-DLB. Actually, some dementia patients with posterior cortical atrophy (PCA) syndrome, which usually includes simultanagnosia together with symptoms of Balint's syndrome (ocular apraxia, optic ataxia, a defective ability to estimate distances, and a depth perception deficit), also suffer from DLB neuropathology [95–98]. Moreover, some visuoperceptual and visuospatial tests (i.e., the visual counting task and form perception task), which are frequently impaired in DLB cases [25, 27], could be sensitive to some aspect of simultanagnosia, even if they were not directly aimed at detecting it. Nonetheless, in our case report, simultanagnosia was highlighted early, during the MCI phase. Simultanagnosia was directly examined with tests purposely aimed to assess it [94, 99]. Further reports of simultanagnosia in MCI-DLB are necessary to confirm its relevance as one of the early cognitive impairments in DLB. We cannot exclude the fact that this feature represents an idiosyncrasy of our patient. Nonetheless, simultanagnosia was linked to the damage of some brain regions (medial parts of the occipital-parietal junction, the cuneus, and inferior intraparietal sulcus, along with damage to the underlying white matter tracts) [94, 100] compatible with the occipital and parietal brain atrophy and hypometabolism usually found in DLB [101, 102]. Our suggestion to assess simultanagnosia in suspected MCI-DLB cases could be important for clinicians, considering that tests for simultanagnosia are somewhat specific and are most likely not included in standard neuropsychological batteries for MCI.

## 5. Conclusions

In summary, our case study confirms the relevance of hallucinations as an early predictor of DLB and better defines their specific features in this class of patients. Moreover, it suggests that simultanagnosia could be a possible predictor of DLB, in particular, during the MCI phase of the disease. Finally, the specific presentation of hallucination symptoms in the present case strongly supports the hypothesis that hallucinations in the earliest stages of DLB may reflect a narcolepsy-like REM disorder. Indeed, a possible association between hallucinations and visuoperceptual/spatial impairments has been confirmed in the present case at intermediate stages. Although caution should be exercised when making generalizations from results of a single case study, we hope that our findings will promote further discussion and research on these topics.

## Figures and Tables

**Table 1:** Longitudinal follow-up.

	Jul-04	Apr-06	Oct-07	Dec-07	Jan-08	May-08
MMSE	28	29	25	18	15	10
ADL	6	6	4	3	3	2
IADL	7	7	1	1	1	1

MMSE: mini mental state examination.

ADL: activity of daily living.

IADL: instrumental activity of daily living.

**Table 2:** Content of BG's hallucinations.

N	Content
1	BG heard a feeble noise (“POOF”) of something falling on the bed (one of the souls) and then a noise/switch as if someone was crawling into bed (“WHOOSH”). After a while, she heard her husband's whisper (“OOOOOH”).
2	As before but without her husband's voice.
3	She heard the same feeble noise of something falling on the bed (“POOF”) and perceived a vibration at the foot of the bed (“such as children jumping at the foot of the bed”).
4	She perceived a loud vibration at the foot of the bed (“the bed went up and down…I felt like I was jumping…just like during an earthquake”).
5	She perceived a vibration at the foot of the bed and then she saw a little light on the fingertip of one of the souls, pointing out the way to the bathroom.
6	A kitchen seat cushion seemed to be swollen, and the patient felt (with her hand) something moving inside and knocking twice.
7	She felt, for a second time, something moving inside the kitchen seat cushion (which she brought with her in the bedroom) (“such as bones”), and then she heard a noise (“STUMP”) as if someone fell down from the chair, which had a short leg. After a while, she heard 6 or 7 smacks, such as her husband used to do.
8	She heard people whispering and then she perceived the same vibration at the foot of the mattress. In this case, the vibration was strong enough to make a loud noise such as a motor scooter.
9	She heard her husband's voice saying turn off the fan (in a dialect form: “smursa smursa smursa”).
10	She saw a child in a plastic see-through bag getting out of bed, stopping, and then getting back into bed (“It was something about one meter high, baby-hands and baby-feet, a round little head, a pretty face, it looked at me, it was a child, no hair, it was in a see-through bag, hands and feet visible, privates not visible so I wasn't able to single out the sex”).
11	She saw the child again and then heard her daughter's voice saying “mammy.”
12	There were two people (“wandering souls”) talking about her. A man and a woman. The woman said to the man, “she wears a small chain with St. Rita and rosary beads”; he said, “now I go and see.” Then, they got into bed and he said, “Now she's crying.”
13	She saw a red-haired woman who seemed not to be very tall, perhaps because she was in a see-through case, seated on a battered chair in the bathroom. The hallucination tried to get up from the chair to go and see what there was on the toilette but she slipped and fell down. Meanwhile, BG also saw a taller man, standing near the bathroom against the wall dressed in black. Later, she would describe him with a wide-brimmed hat—a large hat—that covered his eyes and his face. The man and the woman are the same who spoke in the previous episode.
14	The man and the woman were talking and commenting on her deeds. She went to the bedroom and got a pool card to play with. “Ecco, lee a lè prunta de andà a giügà” in a Milanese dialect (“It's ready to play”). Then, the patient went back to the kitchen. The man said to the woman that the patient went to the kitchen and the woman said, “I want to see.”
15	She felt that the pillow in the armchair was firm and this fact was bizarre because she used it every day. Thus, she thought that it had something inside it, a soul. Then, the pillow seemed to her to have become soft as the soul left.
16	She saw some grooves throughout the bed (3 or 4) and the pillowcase was crinkled. Later, she spoke about the grooves throughout the bed and about the cover that was rolled up, as if the souls went through them. Those signs meant people went through them.
17	She felt a fist on her back coming out from the mattress while she was lying on her bed, such as a fist or a knee or an arm.
18	She heard the voice of an ancient man, 200–300 years old (a croaky voice), who was speaking, but she did not remember anything that he said. Then, the cover slipped off her shoulders and the man put the cover back on her.
19	She heard the voice of a young man from Emilia who said to her, while touching her shoulders, “what are you saying?”. The patient said, “Why? What did I say?” And he answered, “You spoke in your sleep.”
20	One night, the same young man said to her the number 13, but she had never played it and it was drawn three times on the wheel of Venice.
21	The patient had a photo of her husband on the night table, but the souls did not want her to have a photo of her husband. She heard the souls speaking and one of them, pointing to the photo, said in a Milanese dialect, “ah la tegn chi” (“she had it there”). So she took away the photo of her husband and put a photo of Our Lady of Fatima on the night table. Before the voices came, she felt the same vibration at the foot of the bed, which was a sign that souls were coming.
22	She heard the souls talking to each other but she did not understand what they were saying and then she heard a noise like a dead weight falling down. She thought that they had moved one of the souls from the bed and, indeed, the day after, it was no longer there.

**Table 3:** Neuropsychological test results.

Test	BG's raw score	Maximum score	Impairment
General			
Mini mental state examination (MMSE)	29	/30	
Raven coloured progressive matrices	19	/36	
Executive functioning			
Wisconsin card sorting test (WCST)	118	/128	Impaired
Trail making test (TMT)			
Part A	128 s	(mean 89.20 s)	Impaired
Part B	378 s	(mean 211.3 s)	Impaired
Weigl test	2	/15	Impaired
Cognitive estimates (bizarre errors)	7	/21	Impaired
Verbal fluency (phonological one)	30	(mean 25.96)	
Verbal fluency (semantic one)	17	(mean 11.78)	
Digit span backward	4		
Memory			
Short-term memory			
Digit span forward	5	(mean 5.2)	
Corsi's block tapping test	4	(mean 3.79)	
Anterograde memory			
Prose memory	7	/16	
Rey-Osterrieth complex figure recall	11.5	/36	
Paired associated word couples	8.5	/22.5	
Corsi supraspan	6.18	/29.16	
Semantic memory			
Picture to picture matching task	28	/30	
Words to words matching task	30	/30	
Vocabulary test	15	/15	
Visual attention			
Bell's test	33	/35	
Digit cancellation test	39	/60	
Language			
Aachener aphasia test (AAT)			
Token test	3	/50	
Repetition	140	/150	
Written language	86	/90	
Naming	109	/120	
Comprehension	106	/120	
Praxis			
Orofacial apraxia	20	/20	
Ideomotor apraxia (right)	72	/72	
Ideomotor apraxia (left)	72	/72	
Visual perception			
Birmingham Object Recognition Battery			
Minimal feature view task	25	/25	
Object decision task	100	/128	Impaired
Associative match task	30	/30	
Discrimination of scribbles	32	/32	
Picture naming	68	/80	
Navon's letters	30	/60	Impaired
Visuospatial and constructional functions			
Dot counting (VOSP)	9	/10	
Copy of geometrical figures	12	/14	
Rey-Osterrieth complex figure (copy)	23.5	/36	Impaired
Finger localization test	18	/24	

Impaired performances in the impairment column correspond to scores below the 5th percentile.

VOSP: Visual Object Space Perception Battery.
